# A Validation Study of a Commercial Wearable Device to Automatically Detect and Estimate Sleep

**DOI:** 10.3390/bios11060185

**Published:** 2021-06-08

**Authors:** Dean J. Miller, Gregory D. Roach, Michele Lastella, Aaron T. Scanlan, Clint R. Bellenger, Shona L. Halson, Charli Sargent

**Affiliations:** 1 The Appleton Institute for Behavioural Science, CQUniversity, Adelaide, SA 5043, Australia; greg.roach@cqu.edu.au (G.D.R.); m.lastella@cqu.edu.au (M.L.); 2 Human Exercise and Training Laboratory, CQUniversity, Rockhampton, QLD 4700, Australia; a.scanlan@cqu.edu.au; 3 Alliance for Research in Exercise, Nutrition and Activity (ARENA), Allied Health and Human Performance, University of South Australia, Adelaide, SA 5001, Australia; clint.bellenger@unisa.edu.au; 4 South Australian Sports Institute, Adelaide, SA 5001, Australia; 5 School of Behavioural and Health Sciences, Australian Catholic University, Brisbane, QLD 4014, Australia; shona.halson@acu.edu.au

**Keywords:** consumer sleep technology, wearables, PSG, sleep staging, sleep monitoring, sleep quality

## Abstract

The aims of this study were to: (1) compare actigraphy (ACTICAL) and a commercially available sleep wearable (i.e., WHOOP) under two functionalities (i.e., sleep auto-detection (WHOOP-AUTO) and manual adjustment of sleep (WHOOP-MANUAL)) for two-stage categorisation of sleep (sleep or wake) against polysomnography, and; (2) compare WHOOP-AUTO and WHOOP-MANUAL for four-stage categorisation of sleep (wake, light sleep, slow wave sleep (SWS), or rapid eye movement sleep (REM)) against polysomnography. Six healthy adults (male: *n* = 3; female: *n* = 3; age: 23.0 ± 2.2 yr) participated in the nine-night protocol. Fifty-four sleeps assessed by ACTICAL, WHOOP-AUTO and WHOOP-MANUAL were compared to polysomnography using difference testing, Bland–Altman comparisons, and 30-s epoch-by-epoch comparisons. Compared to polysomnography, ACTICAL overestimated total sleep time (37.6 min) and underestimated wake (−37.6 min); WHOOP-AUTO underestimated SWS (−15.5 min); and WHOOP-MANUAL underestimated wake (−16.7 min). For ACTICAL, sensitivity for sleep, specificity for wake and overall agreement were 98%, 60% and 89%, respectively. For WHOOP-AUTO, sensitivity for sleep, wake, and agreement for two-stage and four-stage categorisation of sleep were 90%, 60%, 86% and 63%, respectively. For WHOOP-MANUAL, sensitivity for sleep, wake, and agreement for two-stage and four-stage categorisation of sleep were 97%, 45%, 90% and 62%, respectively. WHOOP-AUTO and WHOOP-MANUAL have a similar sensitivity and specificity to actigraphy for two-stage categorisation of sleep and can be used as a practical alternative to polysomnography for two-stage categorisation of sleep and four-stage categorisation of sleep.

## 1. Introduction

Polysomnography (PSG) is the gold standard method of objectively assessing sleep [1]. However, PSG is expensive, time consuming and impractical in some field settings [2]. The most accepted alternative to PSG is research-grade actigraphy [3]. Actigraphy uses algorithms based on the association of movement and wakefulness, allowing for an objective measurement of sleep and wake [4]. However, acquiring accurate actigraphy data can be cumbersome and requires certain resources and expertise (e.g., proprietary software, reliance of self-reported sleep times, retrospective data extraction) [3,5]. Modern commercial wearable technology provides a user-friendly, accessible alternative to PSG and actigraphy that provides easily accessible aggregated sleep data [3,5]. Unlike actigraphy, which relies solely on accelerometer-based movement detection to measure sleep and wake, sleep wearable technology utilises accelerometers and heart rate tracking technology (photoplethysmography) to provide two-stage categorisation and four-stage categorisation of sleep [3]. Photoplethysmography provides a convenient and accurate indication of autonomic nervous system status by measuring heart rate variability [6].

While providing a convenient alternative to PSG for measuring sleep, the use of actigraphy relies on self-report data from the user (i.e., bedtime, get up time) for retrospective manual adjustment of bed and wake times by the researcher [3]. In comparison, most commercial sleep wearables estimate sleep under two functionalities: auto-detection (i.e., automatic detection of sleep onset and sleep offset) or manual adjustment (i.e., manual input of bed and wake times after a sleep period). The distinction between auto-detected and manually adjusted sleep records is important as they are two different methods of measuring sleep. However, most validation studies for sleep wearables have either analysed manually adjusted data, [5,7,8] or have not reported their methods of data acquisition (i.e., auto-detection versus manual) [9,10,11]. For manually adjusted data validations, the adjustments are performed by researchers in a controlled laboratory setting [5,12]. Therefore, the accuracy of sleep wearables in situations where manual adjustment of sleep times is performed by the user may vary. In this context, actigraphy and sleep wearables utilising a manual adjustment function are subject to compliance of wearing the device and accurately reporting bed and wake times. Compliance for self-report measures is a common obstacle in acquiring accurate data in clinical settings [13] and elite sport [14]. Therefore, sleep wearables that are capable of accurately auto-detecting sleep, and therefore eliminating the non-compliance of users reporting bed and wake times, would provide an attractive alternative for measuring sleep in non-laboratory settings.

The WHOOP strap is a sleep wearable capable of estimating sleep [5]. When using manually adjusted sleep records (i.e., WHOOP-MANUAL), the WHOOP strap has been validated as an alternative for two-stage categorisation of sleep (i.e., sleep and wake) and four-stage categorisation of sleep (i.e., wake, light sleep, slow wave sleep (SWS), rapid eye movement sleep (REM)) when PSG is impractical [5]. However, the ability of the WHOOP strap to automatically detect (i.e., WHOOP-AUTO) and categorise two-stage sleep and four-stage sleep has not been examined. Therefore, the two aims of this study were to: (1) compare the ability of WHOOP-AUTO, WHOOP-MANUAL and research grade actigraphy (ACTICAL) for two-stage categorisation of sleep against PSG, and; (2) compare the ability of WHOOP-AUTO and WHOOP-MANUAL for four-stage categorisation of sleep against PSG.

## 2. Methods

### 2.1. Participants

Six healthy, young adults (male: *n* = 3; female: *n* = 3; age: 23.0 ± 2.2 yr; height: 170.5 ± 7.2 cm; weight: 65.8 ± 3.6 kg) participated in this study. Participants were excluded if they reported any existing medical conditions or sleep disorders or had a recent history of shift work and/or transmeridian travel. The study was approved by the Central Queensland University Human Research Ethics Committee.

### 2.2. Laboratory Setting

The study was conducted in a purpose-built accommodation suite at the Appleton Institute for Behavioural Science, Central Queensland University, Adelaide, Australia. The suite is sound-attenuated, free from external environmental cues and simultaneously houses six participants with private bedrooms and bathrooms.

### 2.3. Design

**Figure 1:**
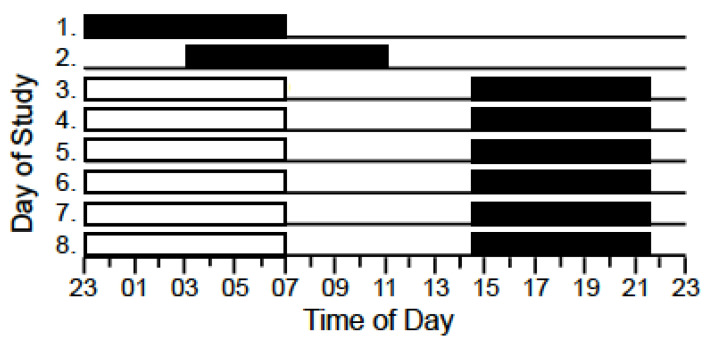
Illustration of the study design. Black horizontal bars indicate sleep opportunities. White horizontal bars indicate simulated work periods. *Y*-axis: “Day of Study”. *X*-axis: “Time of Day”.

Data were collected as part of a larger experimental study. Participants lived in a sleep laboratory for ten consecutive nights/days and were given sleep opportunities of varying durations. Participants were given 9 h sleep opportunities on nights 1 (23:00–08:00 h) and 2 (03:00–12:00 h); 7 h sleep opportunities on days 3–8 (14:30–21:30 h) and day 9 (08:30–15:30 h; Figure 1). Participants completed simulated work shifts on days 3–9 and performed sedentary tasks during free time (i.e., reading, watching movies; Figure 1). Data were collected between the 16th and the 25th of July 2019.

### 2.4. Measures and Procedure

Sleep was measured using PSG. A standard montage of electrodes was attached to the face and scalp of participants (i.e., C4-M1, F4-M1, O2-M1), including two electro-oculograms (i.e., left/right outer canthus) and a submental electromyogram [15]. PSG data were recorded directly to data acquisition, storage, and analysis systems (Grael, Compumedics; Melbourne, Victoria, Australia). PSG records were manually scored in 30-s epochs by an experienced registered polysomnographic technician in compliance with standard criteria [16]. The commercially available wearable device used in this study was the WHOOP strap (Generation 2.0, CB Rank, Greater Boston, New England). The research grade activity monitor used in this study was the Actical Z-series (ACTICAL; Mini-Mitter Philips Respironics, Inc., Bend, OR, USA) [17]. Participants wore the WHOOP strap and ACTICAL on their non-dominant wrist, with the WHOOP strap placed 1 cm above the wrist bone—proximal to the ACTICAL. Prior to the study, clock time was manually synchronised on all devices (i.e., laboratory computers, ACTICAL, mobile devices running the WHOOP iOS application).

Data for automatically detected sleep (WHOOP-AUTO) and for manually adjusted sleep (WHOOP-MANUAL) were provided by the manufacturer for comparison to PSG. WHOOP-AUTO data were provided first to ensure that the manufacturer was blind to sleep times. Once WHOOP-AUTO data were received, the start and end times of each sleep opportunity were manually entered by a researcher into the WHOOP iOS application and the manually adjusted data (WHOOP-MANUAL) were then provided by the manufacturer. Epoch-by-epoch ACTICAL data were obtained using accompanying software (30-s epochs, medium sensitivity threshold; Actiware version 3.4; Mini-Mitter Philips Respironics, Inc.) [17].

The following sleep variables were collected during the study:

- Total sleep time (TST): the sum of minutes spent in any stage of sleep (N1, N2, N3, REM).
- Wake: the sum of minutes spent awake during the sleep opportunity.
- Light sleep: the sum of minutes spent in stage N1 or N2 sleep.
- Slow wave sleep (SWS): the sum of minutes spent in stage N3 sleep.
- Rapid eye movement sleep (REM): the sum of minutes spent in stage REM.
- Sleep onset latency (SOL): the duration of time from lights out to the first epoch of any stage of sleep.

PSG and WHOOP-MANUAL provided records of all the above variables. WHOOP-AUTO provided records of all the above variables, except for sleep onset latency. ACTICAL provided records of total sleep time and wake only.

To ensure that the WHOOP-AUTO, WHOOP-MANUAL and ACTICAL data were properly aligned to PSG data for each sleep record, agreement was calculated for offset adjustments of ±3 min in 30-s increments [18]. In all cases, agreement was not substantially improved by applying an offset, so all subsequent analyses were based on unadjusted data.

## 3. Data Analysis

Differences in TST, wake, light sleep, SWS and REM between PSG, WHOOP-AUTO and WHOOP-MANUAL were tested using separate General Linear Mixed Models (R package lme4; R Core Team, 2016). Differences in TST and wake time between PSG and ACTICAL were analysed using separate General Linear Mixed Models (R package lme4; R Core Team, 2016). A random intercept for participants was included in each model to account for intraindividual dependencies and interindividual heterogeneity.

Agreement between PSG and WHOOP-AUTO, WHOOP-MANUAL and ACTICAL was tested using the Bland–Altman limits of agreement method for repeated measurements [19]. For each sleep variable, the difference between PSG and WHOOP-AUTO, WHOOP-MANUAL and ACTICAL (i.e., bias) and the 95% limits of agreement (i.e., bias ± 1.96*SD) were plotted. Each plot was examined for heteroscedasticity and proportional bias using the Breusch–Pagan test and least ordinary squares regression, respectively. If proportional bias or heteroscedasticity was present, the bias and 95% limits of agreement were adjusted accordingly [20].

To conduct epoch-by-epoch comparisons for two-stage categorisation of sleep, WHOOP-AUTO, WHOOP-MANUAL and ACTICAL data were arranged in 30-s epochs and aligned with the corresponding PSG record. The following measures were then calculated for WHOOP-AUTO, WHOOP-MANUAL and ACTICAL:

- Sensitivity: the percentage of PSG-determined sleep epochs correctly identified as sleep by each method;
- Specificity: the percentage of PSG-determined wake epochs correctly identified as wake by each method;
- Agreement: the percentage of PSG-determined sleep and wake epochs correctly identified as sleep or wake by each method.

To conduct epoch-by-epoch comparisons for four-stage categorisation of sleep, WHOOP-AUTO and WHOOP-MANUAL data were arranged in 30-s epochs and aligned with the corresponding PSG record. The following measures were then calculated for WHOOP-AUTO and WHOOP-MANUAL:

- Sensitivity for wake: the percentage of PSG-determined wake epochs correctly identified as wake by each method;
- Sensitivity for light sleep: the percentage of PSG-determined N1 and N2 epochs correctly identified as light sleep by each method;
- Sensitivity for SWS: the percentage of PSG-determined N3 epochs correctly identified as SWS by each method;
- Sensitivity for REM: the percentage of PSG-determined REM epochs correctly identified as REM by each method;
- Agreement: the percentage of PSG-determined N1, N2, REM, and wake epochs correctly identified as light sleep, deep sleep, REM, or wake by each method.

Cohen’s kappa (κ) was calculated to evaluate agreement between PSG and WHOOP-AUTO, WHOOP-MANUAL and ACTICAL beyond what could be expected by chance [21]. Agreement was interpreted against recommended guidelines as: *slight* agreement = 0–0.20; *fair* agreement = 0.21–0.40; *moderate* agreement = 0.41–0.60; *substantial* agreement = 0.61–0.80; *almost perfect* agreement = 0.81–0.99; and *perfect* agreement = 1 [22]. Intraclass correlation coefficients were calculated to assess the reliability of WHOOP-AUTO, WHOOP-MANUAL and ACTICAL for two- and four-stage categorisation of sleep [23]. Intraclass correlation coefficients were interpreted against recommended guidelines as: “*poor*” = <0.40; “*fair*” = 0.40–0.59; “*good*” = 0.60–0.74; and “*excellent*” = 0.75–1.00 [24].

Aggregated data were collated from previous studies to compare WHOOP-AUTO, WHOOP-MANUAL and ACTICAL, respectively, against previous validations of sleep wearables [5,7,9,10,11,12,25,26,27,28,29,30,31].

## 4. Result

Data acquired using WHOOP-AUTO (*n* = 54), WHOOP-MANUAL (*n* = 54) and ACTICAL (*n* = 54) were included in the analyses for comparison to PSG. No data were lost and WHOOP-AUTO correctly identified 100% of the 54 sleep opportunities.

For two-stage categorisation of sleep, there was no significant difference between WHOOP-AUTO and PSG for TST or wake time (Table 1). Epoch-by-epoch data showed high sensitivity and moderate specificity for WHOOP-AUTO against PSG (Table 2). Cohen’s kappa coefficient indicated *moderate* agreement (κ = 0.44) between the WHOOP-AUTO and PSG for two-stage categorisation of sleep [22]. Intraclass coefficient correlation indicated *fair* reliability (0.45) between WHOOP-AUTO and PSG for two-stage categorisation of sleep.

**Figure 2:**
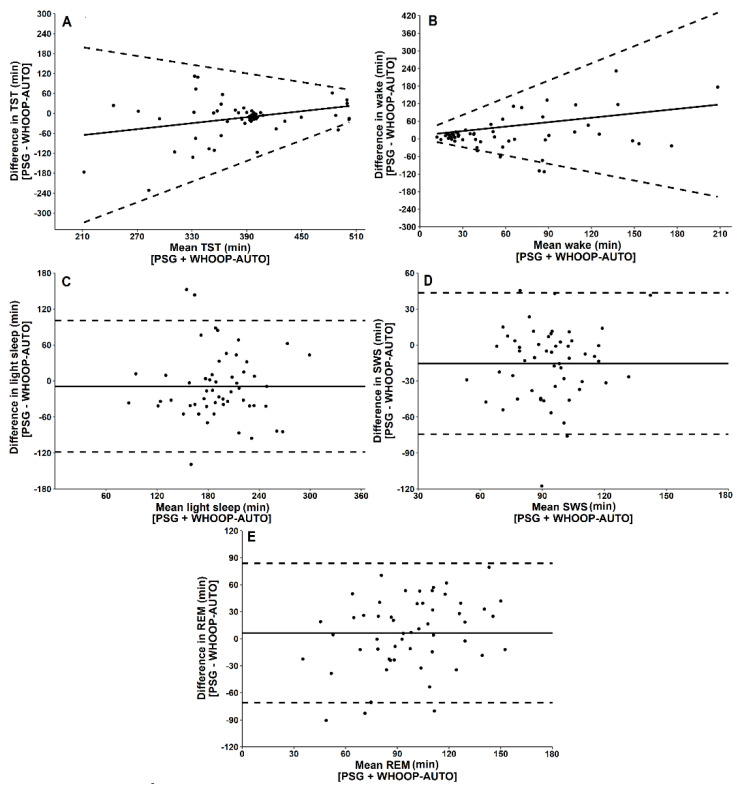
Bland–Altman plots for WHOOP-AUTO and PSG-derived measures of (**A**) total sleep time (TST), (**B**) wake time, (**C**) light sleep, (**D**) slow wave sleep (SWS), (**E**) rapid eye movement sleep (REM). Data points represent one sleep opportunity. The x-axes represent the mean of the values obtained from WHOOP-AUTO and PSG. The y-axes represent the difference between the values, such that positive values indicate that WHOOP-AUTO overestimates relative to PSG and negative values indicate that WHOOP-AUTO underestimates relative to PSG. Solid horizontal lines indicate the mean bias from PSG, and broken lines indicate the 95% limits of agreement (±1.96 standard deviations) [20].

For two-stage categorisation of sleep, there was no significant difference between WHOOP-MANUAL and PSG for TST, but WHOOP-MANUAL significantly underestimated wake time compared to PSG (Figure 2; Table 1). Epoch-by-epoch data showed high sensitivity, but low specificity compared to PSG (Table 2). Cohen’s kappa coefficient indicated *moderate* agreement (κ = 0.48) between the WHOOP-MANUAL and PSG for two-stage categorisation of sleep. Intraclass coefficient correlation indicated *fair* reliability (0.48) between WHOOP-MANUAL and PSG for two-stage categorisation of sleep.

**Figure 3:**
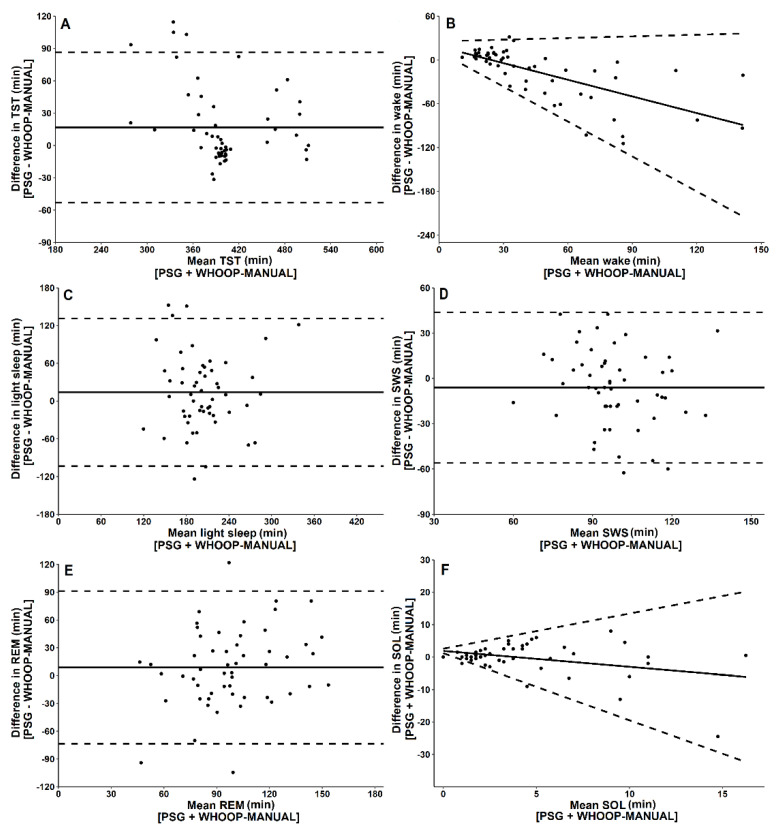
Bland–Altman plots for WHOOP-MANUAL and PSG-derived measures of (**A**) total sleep time (TST), (**B**) wake time, (**C**) light sleep, (**D**) slow wave sleep (SWS), (**E**) rapid eye movement sleep (REM) and (**F**) sleep onset latency (SOL). Data points represent one sleep opportunity. The x-axes represent the mean of the values obtained from WHOOP-MANUAL and PSG. The y-axes represent the difference between the values, such that positive values indicate WHOOP-MANUAL overestimates relative to PSG and negative values indicate WHOOP-MANUAL underestimates relative to PSG. Solid horizontal lines indicate the mean bias from PSG, and broken lines indicate the 95% limits of agreement (±1.96 standard deviations) [20].

For four-stage categorisation of sleep, there was no significant difference between WHOOP-AUTO and PSG for TST, wake time, light sleep or REM. WHOOP-AUTO significantly underestimated SWS and overestimated sleep onset latency (Figure 3; Table 1). There was moderate overall agreement for four-stage categorisation of sleep between PSG and WHOOP-AUTO and moderate sensitivity for wake, light sleep, SWS and REM (Table 2). Cohen’s kappa coefficient indicated moderate agreement (κ = 0.47) between WHOOP-AUTO and PSG for four-stage categorisation of sleep [22]. Intraclass coefficient correlation indicated *fair* reliability (0.48) between WHOOP-AUTO and PSG for four-stage categorisation of sleep.

A four-stage error matrix comparing WHOOP-AUTO and PSG is presented in Table 3. When WHOOP-AUTO misclassifies wake, it classifies it as light sleep. When WHOOP-AUTO misclassifies light sleep, it classifies it as either wake or REM. When WHOOP-AUTO misclassifies SWS, it classifies it as light sleep. When WHOOP-AUTO misclassifies REM, it classifies it as light sleep. Bland–Altman plots comparing WHOOP-AUTO to PSG for each sleep variable are depicted in Figure 2. Proportional bias (i.e., whether the differences between a device and PSG change as a function of duration) and heteroscedasticity (i.e., whether variance changes as a function of duration) were present for TST, wake time, and sleep onset latency, but not for light sleep, SWS or REM.

**Figure 4:**
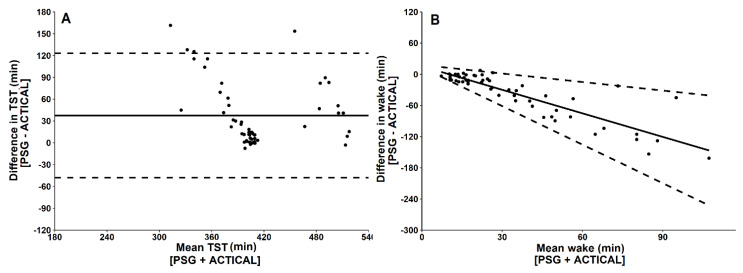
Bland–Altman plots for ACTICAL and PSG-derived measures of (**A**) total sleep time (TST) and (**B**) wake time. Data points represent one sleep opportunity. The x-axes represent the mean of the values obtained from ACTICAL and PSG. The y-axes represent the difference between the values, such that positive values indicate that ACTICAL overestimates relative to PSG and negative values indicate that ACTICAL underestimates relative to PSG. Solid horizontal lines indicate the mean bias from PSG, and broken lines indicate the 95% limits of agreement (±1.96 standard deviations) [20].

ACTICAL significantly overestimated TST and underestimated wake when compared to PSG (Figure 4; Table 1). For two-stage categorisation of sleep, ACTICAL had high sensitivity (i.e., ability to detect sleep) and moderate specificity (i.e., ability to detect wake; Table 2). Cohen’s kappa coefficient for two-stage categorisation of sleep (κ = 0.23) indicated *fair* agreement between ACTICAL and PSG. Intraclass coefficient correlation indicated *poor* reliability (0.26) between ACTICAL and PSG for two-stage categorisation of sleep. Bland–Altman plots comparing ACTICAL to PSG for each sleep variable are depicted in Figure 4.

For four-stage categorisation of sleep, there was no significant difference between WHOOP-MANUAL and PSG for TST, light sleep, SWS, REM or sleep onset latency. WHOOP-MANUAL significantly underestimated wake time compared to PSG (Table 1). There was *moderate* overall agreement for four-stage categorisation of sleep, *moderate* sensitivity for light sleep, SWS and REM, and *low* sensitivity for wake time between PSG and WHOOP-MANUAL (Table 2). Cohen’s kappa coefficient indicated *moderate* agreement (κ = 0.49) between WHOOP-MANUAL and PSG for four-stage categorisation of sleep [22]. Intraclass coefficient correlation indicated *fair* reliability (0.47) between WHOOP-MANUAL and PSG for two-stage categorisation of sleep.

A four-stage error matrix comparing the WHOOP-MANUAL and PSG is presented in Table 4. When WHOOP-MANUAL misclassifies wake, it classifies it as light sleep. When WHOOP-MANUAL misclassifies light sleep, it classifies it as REM. When WHOOP-MANUAL misclassifies SWS, it classifies it as light sleep. When WHOOP-MANUAL misclassifies REM, it classifies it as light sleep. Bland–Altman plots comparing WHOOP-MANUAL to PSG for each sleep variable are depicted in Figure 3. Proportional bias and heteroscedasticity were present for wake time and sleep onset latency, but not for TST, light sleep, SWS or REM.

## 5. Discussion

The two aims of this study were to: (1) compare WHOOP-AUTO, WHOOP-MANUAL and research grade actigraphy (ACTICAL) for two-stage categorisation of sleep against PSG, and; (2) compare WHOOP-AUTO and WHOOP-MANUAL for four-stage categorisation of sleep against PSG.

### 5.1. Two-Stage Categorisation of Sleep

Actigraphy is commonly utilised as an objective measure of sleep and wake by practitioners [3,30,32]. However, the process of acquiring sleep data using actigraphy requires certain expertise and is usually a retrospective analysis of an extended data collection period—rather than the immediate day-by-day data that are provided by modern sleep wearables. The accuracy of actigraphy and sleep wearables utilising a manual adjustment function is subject to the compliance of the user wearing the device and accurately reporting bed and wake times. In this context, it is important to compare the performance of actigraphy (i.e., ACTICAL) to modern sleep wearables that can automatically detect sleep and provide easily accessible data (e.g., the WHOOP strap).

Regarding the two-stage detection of sleep, WHOOP-AUTO, WHOOP-MANUAL and ACTICAL had high sensitivity for sleep (97, 90 and 98%, respectively), but WHOOP-MANUAL had lower specificity for wake (45%) than WHOOP-AUTO (60%) and ACTICAL (60%). Chance-corrected agreement was *fair* for ACTICAL (κ = 0.23) and *moderate* for WHOOP-AUTO (κ = 0.44) and WHOOP-MANUAL (κ = 0.48). Intraclass correlation coefficients showed that WHOOP-AUTO (0.45) and WHOOP-MANUAL (0.48) had *fair* reliability for two-stage classification of sleep, compared to *poor* reliability for ACTICAL (0.26). Comparisons of reliability based on intraclass correlations should be made across devices within the same study as there is no clear threshold at which a device can be considered “valid” [3]. It should be noted that a previous validation study conducted in the same laboratory found WHOOP-MANUAL to have a 51% specificity for wake when compared to PSG [5]. These findings support a previous validation of WHOOP-MANUAL two-stage categorisation of sleep [5] and provide novel support for WHOOP-AUTO as a practical alternative for two-stage categorisation of sleep in the absence of PSG.

### 5.2. Four-Stage Categorisation of Sleep

For four-stage categorisation of sleep, WHOOP-AUTO and WHOOP-MANUAL had similar overall agreement (63% and 62%, respectively) and sensitivity to light sleep (61% and 67%, respectively), SWS (63% and 61%, respectively) and REM (66% and 66%, respectively). Chance-corrected agreement for four-stage categorisation of sleep was *moderate* for WHOOP-AUTO (κ = 0.47) and WHOOP-MANUAL (κ = 0.49). As a reference point, the chance-corrected agreement between expert sleep scorers independently scoring a common set of PSG records was substantial rather than perfect (κ = 0.78) [33]. These results support a previous validation of WHOOP-MANUAL to measure four-stage and provide the first validation of WHOOP-AUTO as a practical alternative for four-stage categorisation of sleep in the absence of PSG. The main disparity between WHOOP-AUTO and WHOOP-MANUAL for four-stage categorisation of sleep compared to PSG was that WHOOP-AUTO exhibited 16% higher sensitivity for wake compared to WHOOP-MANUAL. However, WHOOP-MANUAL can provide an accurate measure of onset latency (Table 1). Depending on the variable of interest, practitioners seeking to utilise the WHOOP strap to measure sleep can selectively utilise WHOOP-AUTO or WHOOP-MANUAL functions. For example, in situations where the WHOOP strap is utilised for two-stage or four-stage categorisation of sleep for sleep opportunities between 7 and 9 h, WHOOP-AUTO appears to be the more practical, better performing function. However, given that WHOOP-MANUAL utilises a reference point for when an individual begins to attempt sleep, it should be used in situations where sleep onset latency is the variable of interest.

The difference for estimating wake between WHOOP-AUTO and WHOOP-MANUAL in this study highlights the need for future validation research to report the ability of sleep wearables to measure a range of sleep measures under auto-detection and manual function. Previous validation studies for consumer sleep wearables do not explicitly report whether data were acquired using the automatic detection of sleep or manual entering of sleep times [7,8,9,10,11], thus limiting practitioners’ ability to best utilise sleep wearables to measure specific sleep variables. Overall, the findings of this study suggest that WHOOP-AUTO and WHOOP-MANUAL may be used as a practical alternative for two-stage categorisation of sleep and four-stage categorisation of sleep when PSG is not available.

### 5.3. Comparison to Other Sleep Wearables

**Figure 5:**
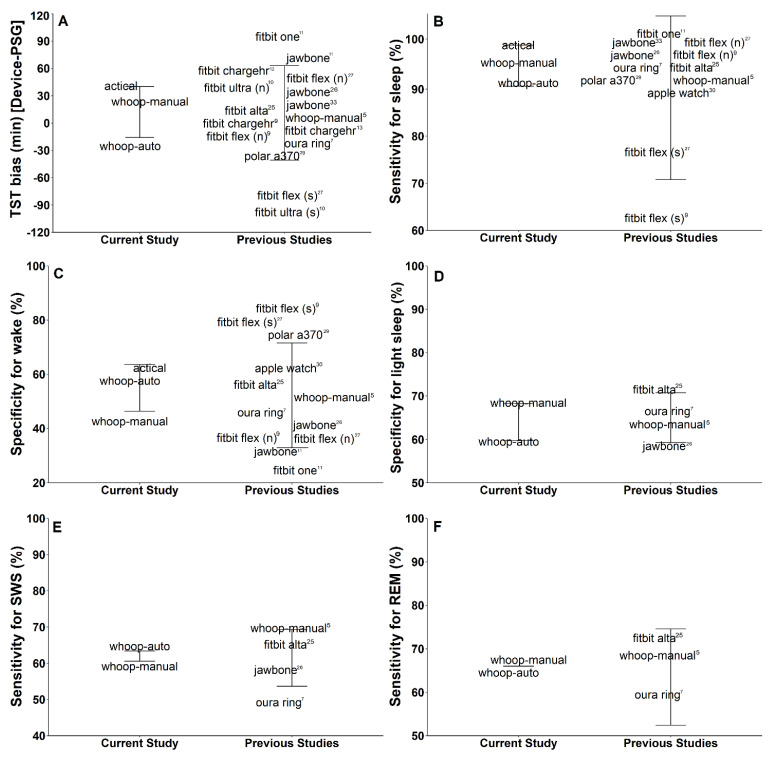
Performance of ACTICAL, WHOOP-AUTO, WHOOP-MANUAL and other sleep wearables for (**A**) total sleep time bias (TST), (**B**) sensitivity for sleep, (**C**) specificity for wake, (**D**) sensitivity for light sleep, (**E**) sensitivity for slow wave sleep (SWS) and (**F**) sensitivity for rapid eye movement sleep (REM). Error bars represent standard deviation. Fitbit Flex (N); Fitbit Flex with normal sensitivity, Fitbit Flex (S); Fitbit Flex with high sensitivity. Superscript numbers represent respective validation studies [5,7,9,10,11,12,25,26,27,28,29,30,31].

Due to an increase in consumer devices providing measures of sleep, it is important to conduct cross-device comparisons. Ideally, within-study comparisons like in the present study should be made to provide meaningful comparison. However, interpretations of cross-study comparisons can be made with consideration to differences in study methodologies (i.e., sleep opportunity, sample, sleep environment). A comparison of the performance of WHOOP-AUTO, WHOOP-MANUAL and ACTICAL, respectively, to previous sleep wearable validations can be seen in Figure 5 [5,7,9,10,11,12,25,26,27,28,29,30,31].

The WHOOP strap, in both automatic and manual functions, fell within the standard deviation for TST bias, sensitivity for sleep, specificity for wake, sensitivity for light sleep, sensitivity for SWS, and sensitivity for REM compared to previous validations (Figure 5). Previous validations of sleep wearables have shown that there is an apparent “trade-off” between sensitivity and specificity [3], such that higher sensitivity may result in decreased specificity, and vice versa. For example, a validation study conducted with the Fitbit One had high sensitivity but had low specificity compared to PSG (Figure 5) [10]. Compared to the WHOOP strap, other sleep wearables have shown higher sensitivity to individual sleep stages (Figure 5). However, both WHOOP-AUTO and WHOOP-MANUAL appear to be consistent across all four sleep stages and do not seem to exhibit a large “trade-off” between sensitivities for all sleep stages.

According to the methodologies of previous studies, WHOOP-AUTO provides the only epoch-by-epoch comparison to PSG using sleep auto-detection [7,9,10,11,12,25,26,27]. From a practical perspective, WHOOP-AUTO provides a measure of sleep comparable to manually adjusted data and eliminates the risk of non-compliance for entering bed times. Overall, the findings of this validation study suggest that the WHOOP strap, under both automatic and manual detection of sleep, performs well in comparison to other commercially available sleep wearables.

### 5.4. Boundary Conditions and Future Research

This validation study was conducted on the WHOOP-AUTO and WHOOP-MANUAL functions of the WHOOP strap. The validation of other WHOOP metrics (i.e., heart rate, heart rate variability) was outside of the scope of this project. The algorithms used by WHOOP to score sleep are proprietary, and epoch-by-epoch data are not accessible through the WHOOP smart phone application. Findings should also be interpreted within the boundary conditions of the sleep environment (laboratory), time in bed opportunities (7–9 h) and sample (healthy young adults). Future investigations should validate the WHOOP strap and other sleep wearables with reference to all available functionalities (i.e., auto-detection and manual adjustment) and across a wider range of conditions (sleep opportunities of different lengths, disturbed sleep periods, unhealthy and/or older populations).

## Figures and Tables

**Table 1:** Comparison of sleep variables determined by PSG, WHOOP-AUTO, WHOOP-MANUAL and ACTICAL.

Variable (min)		PSG vs. WHOOP-AUTO			PSG vs. WHOOP-MANUAL			PSG vs. ACTICAL		
	PSG	Bias	AE	F	Bias	AE	F	Bias	AE	F
TST	392.8 (60.7)	−17.8 (61.1)	40.0	1.7	16.7 (35.6)	25.4	2.4	37.6 * (85.6)	38.1	12.2
Wake	53.9 (45.7)	17.8 (61.1)	40.0	2.8	−16.7 * (35.6)	25.4	6.3	−37.6 * (85.6)	38.1	35.1
Light	197.1 (50.8)	−8.9 * (55.9)	43.8	0.8	13.9 (59.9)	47.0	2.0			
SWS	101.4 (21.6)	−15.5 ** (30.1)	24.7	13.1	−6.1 (25.4)	20.7	2.8			
REM	94.3 (28.9)	6.5 (39.5)	33.0	0.9	8.8 (42.0)	33.0	1.9			
SOL	5.3 (5.9)				−0.2 (4.8)	2.8	0.01			

Notes: PSG; polysomnography, AE; absolute error (minutes), F; F-statistic, TST; total sleep time, Wake; wake time; Light; light sleep; SWS; slow wave sleep, REM; rapid eye movement sleep; SOL; sleep onset latency. Negative bias indicates an underestimation of the sleep variable by WHOOP-AUTO, WHOOP-MANUAL and ACTICAL when compared to PSG. * indicates significant difference to PSG with *p* < 0.05; ** indicates significant difference to PSG with *p* < 0.001. Data are mean (*SD*).

**Table 2:** Epoch-by-epoch concordance statistics for WHOOP-AUTO (2-stage and 4-stage categorisation of sleep), WHOOP-MANUAL (2-stage and 4-stage categorisation of sleep) and ACTICAL (2-stage categorisation of sleep) against PSG.

Measure	Value (%)
**2-stage comparison**	
WHOOP-AUTO	
Sensitivity for sleep	90
Specificity for wake	60
Overall agreement	86
WHOOP-MANUAL	
Sensitivity for sleep	97
Specificity for wake	45
Overall agreement	90
ACTICAL	
Sensitivity for sleep	98
Specificity for wake	60
Overall agreement	89
**4-stage comparison**	
WHOOP-AUTO	
Sensitivity for wake	60
Sensitivity for light sleep	61
Sensitivity for SWS	63
Sensitivity for REM	66
Overall agreement	63
WHOOP-MANUAL	
Sensitivity for wake	45
Sensitivity for light sleep	67
Sensitivity for SWS	61
Sensitivity for REM	66
Overall agreement	62

Notes: SWS; slow wave sleep, REM; rapid eye movement sleep.

**Table 3:** Four-stage error matrix for WHOOP-AUTO and PSG.

		WHOOP-AUTO			
	**Stage**	**Wake**	Light sleep	SWS	REM
**PSG**	Wake	60%	26%	1%	12%
	Light sleep	14%	61%	10%	15%
	SWS	6%	28%	64%	2%
	REM	6%	27%	1%	66%

Notes: This matrix presents the percentage of each sleep stage that WHOOP-AUTO has correctly or incorrectly classified compared to PSG. Shaded cells indicate correctly classified sleep. SWS; slow wave sleep, REM; rapid eye movement sleep.

**Table 4:** Four-stage error matrix for WHOOP-MANUAL and PSG.

		WHOOP-MANUAL			
	**Stage**	**Wake**	Light sleep	SWS	REM
**PSG**	Wake	45%	37%	1%	18%
	Light sleep	7%	67%	11%	15%
	SWS	1%	38%	61%	1%
	REM	1%	31%	2%	66%

Notes: This matrix presents the percentage of each sleep stage that the WHOOP-MANUAL has correctly or incorrectly classified compared to PSG. Shaded cells indicate correctly classified sleep. SWS; slow wave sleep, REM; rapid eye movement sleep.

## Data Availability

The datasets generated from the current study are available from the corresponding author on reasonable request.
